# Engineering Au Nanoparticle Arrays on SiO_2_ Glass by Pulsed UV Laser Irradiation

**DOI:** 10.1007/s11468-012-9428-3

**Published:** 2012-08-17

**Authors:** K. Grochowska, G. Śliwiński, A. Iwulska, M. Sawczak, N. Nedyalkov, P. Atanasov, G. Obara, M. Obara

**Affiliations:** 1 Photophysics Department, The Szewalski Institute, Polish Academy of Sciences, 14 Fiszera St, 80-231 Gdańsk, Poland; 2 Institute of Electronics, Bulgarian Academy of Sciences, 72 Tzarigradsko Shousse, 1784 Sofia, Bulgaria; 3 Faculty of Science and Technology, Keio University, 3-14-1 Hiyoshi, Kohoku-ku, Yokohama, 223-8522 Japan

**Keywords:** Au nanoparticles, Laser annealing, Semi-regular nanostructures, Surface plasmon resonance

## Abstract

We study semi-regular arrays of Au nanoparticles (NP) obtained via UV laser irradiation of thin Au films on glass substrate. The NP structures are prepared from films of a thickness up to 60 nm produced by discharge sputtering or pulsed laser deposition, and annealed by nanosecond laser pulses at 266 or 308 nm, respectively, at fluencies in the range of 60–410 mJ/cm^2^. For the rare- and close-packed NP structures, consistent description of optical properties is derived from microscopic observation, measurements of the absorption, and Raman spectra, and modeling of the near-field intensity distributions. The absorption bands centered at 540–570 nm are ascribed to resonant absorption of the surface plasmons. For the band positions, half widths, and intensities, the dependence on the NP shape (partial spheres), size, size distribution, and also excitation energy is observed. The structures are characterized by markedly reduced dephasing times of ∼3 fs. It is shown, that laser annealing of thin Au films provides reliable and cost effective method for controlled preparation of semi-regular NP arrays favorable for photonic applications.

## Introduction

Optical properties of gold nanoparticles evoke much interest due to their potential nonlinear applications such as light harvesting, optical tweezers, and ultrasensitive detection. Particular emphasis is still placed on the issue of the surface plasmon resonance (SPR). The excitation of collective oscillations of the free electrons results in a strong light scattering, absorption, and strengthening of the local electromagnetic field in the close vicinity of the nanoparticles. The SPR depends critically on properties of the metal and on these of the surrounding such as dielectric function and refractive index [1, 2]. Among metals, gold represents one of the most preferred materials because of large enhancement of the local field provided under irradiation in the visible and near-infrared part of the spectrum, high stability of the structures, biocompatibility, corrosion resistance, and sufficiently matured preparation techniques of nanoparticles (NPs) [3–5]. The NP structures produced by techniques based on the thin film annealing seem to be advantageous compared to deposited by the electron or ion beam lithography, chemically or colloidal ones. This is confirmed by comparative studies showing that annealing ensures controllable process performance and results in agglomerate-free NP structures of tunable properties [6–9]. The use of UV laser annealing of thin films for nanostructuring has been originally proposed by Henley et al. [10]. A number of works confirm that this technique is sufficiently flexible to provide conclusive research data and offers unexplored application potential, too [9, 11–13].

This work aims on contribution to the systematic study on semi-regular, size-distributed arrays of partially spherical NPs produced by the pulsed UV laser annealing of thin gold films. Properties of NP arrays obtained in a controlled manner by laser annealing of films deposited by two different techniques are investigated. The effect of particle shape and distribution is discussed for rare- and close-packed structures in view of literature results on ordered and random particle configurations [14]. Results of the structural and spectroscopic characterization and of numerical modeling of the near-field distribution are compared with recently published data [15]. It is shown that consistent description of the semiregular structures can be provided by an appropriate modification to the known, size-dependent correction of the dielectric function.

## Experimental

For preparation of the NP arrays, first the thin Au films were produced from bulk material (Sigma Aldrich; 99.99 % purity) by means of the discharge sputtering (DS) or pulsed laser deposition (PLD) applied as more effective in case of thicker films. The DS was performed in vacuum at ambient temperature and at pressure of 4 × 10^−2^ hPa. Films of a thickness of 10–20 nm were produced on the microscope glass slides (2.5 × 2.5 cm^2^) sonically cleaned in sulfuric acid and acetone bath. The growth rate measured by the microbalance was 7.5 nm/min. For preparation of the Au NP structures the films were irradiated in a vacuum chamber (10^−6^ hPa) by the pulsed Nd:YAG laser (Quantel B) operated at 266 nm with pulse duration and repetition frequency of 6 ns and 2 Hz, respectively. Up to 20 pulses were applied per sample at fixed laser fluencies in the range of 10–412 mJ/cm^2^. The Au films of a larger thickness of 60 nm were produced on the glass by PLD using the XeCl laser (308 nm, 30 ns pulse width) under ambient conditions same as applied during DS. For laser fluence of 1.5 J/cm^2^, the deposition rate was 15 nm/min. The same laser operated at lower fluence was applied for nanostructuring of the 60 nm thick film. For both lasers, the fluence values were selected by variation of the laser spot dimension using optical telescope in the laser beam path.

The inspection of the sample morphology was performed by means of the field emission scanning electron microscope (SEM, EVO-40; Zeiss). For absorbance measurements of the reference samples (glass slides and thin Au films) and of the Au NP structures the spectrophotometer UV 1240 (SHIMADZU) was applied. The μ-Raman spectra were acquired by means of the confocal instrument (InVia, Renishaw) equipped with lasers allowing sample excitation at 514 and 785 nm. The measurements of the surface enhanced Raman signal (SERS) signal were performed at similar laser power for both wavelengths, for 0.09 M solution of Rhodamine 6 G (R6G) in ethanol applied on the prepared structures and dried. Spectra were acquired at microscope magnification of ×50 and averaged over at least five scans.

## Results and Discussion

### Nanoparticle Structures

For the thin films deposited on glass substrates by both the sputtering and PLD techniques, the uniform, fine-grained polycrystalline structures were deduced from SEM images. The microscopic inspection of the films showed correlation of the grain size with film thickness and applied deposition rates for both cases. No film discontinuities and other surface defects were noticed. This indicates that such films as base material for nanostructuring when produced under similar conditions and at the same deposition rates are of similar structure and properties. Thus, it can be expected that nanostructures produced in the next step are independent on the film preparation technique. However, this requires careful selection of the laser annealing parameters (fluence, pulse repetition rate) below threshold values corresponding to film fragmentation. Because of no photochemical processes involved, the different irradiation wavelengths of 266 and 308 nm result in minor only differences of the absorbed laser energy via the wavelength dependent reflection coefficient of Au. Under such conditions, the photothermal effect initiates material melting mainly on the grain boundaries. The initially grainy thin film structures nucleate during laser irradiation and form irregular islands. Prolonged irradiation and coalescence together with poor wetting of the glass by liquid gold leads to growth of particles and production of semi-ordered NP structures [14]. The NP size, size distribution, shape and inter-particle distance are controlled by the initial thickness of the Au film and its irradiation conditions during annealing. The final structure geometry results from the balanced surface tension of the gold interfaces with substrate and surrounding characterized by minimal surface area to volume ratio of the metal.

**Fig. 1:**
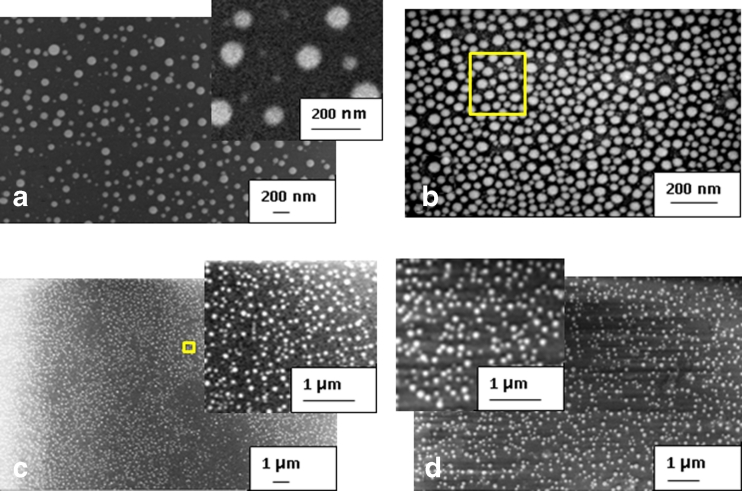
SEM images of Au NP structures on glass obtained from film of a thickness of 10 nm after irradiation at 266 nm (**a**, **c**, **d**) and 60 nm thick film irradiated at 308 nm (**b**); pulse numbers and fixed laser fluencies of: five pulses at 412 mJ/cm^2^ (**a**), 20 at 130 mJ/cm^2^ (**b**), 15 at 60 mJ/cm^2^ (**c**), and 100 mJ/cm^2^ (**d**) were applied; *insets* in **a**, **c**, and **d** are scaled from original images for comparison of particle distributions shown in **a**, **b** and **c**, **d**, respectively; in **b** and **c**, areas of calculated near field intensity distributions are marked

The laser annealing of the sputtered and PL deposited thin Au films results in the semi-regular NP structures shown in Fig. 1. For arrays in Fig. 1a, c, d obtained from 10 nm thick films, the top views reveal NPs of circular symmetry.

**Fig. 2:**
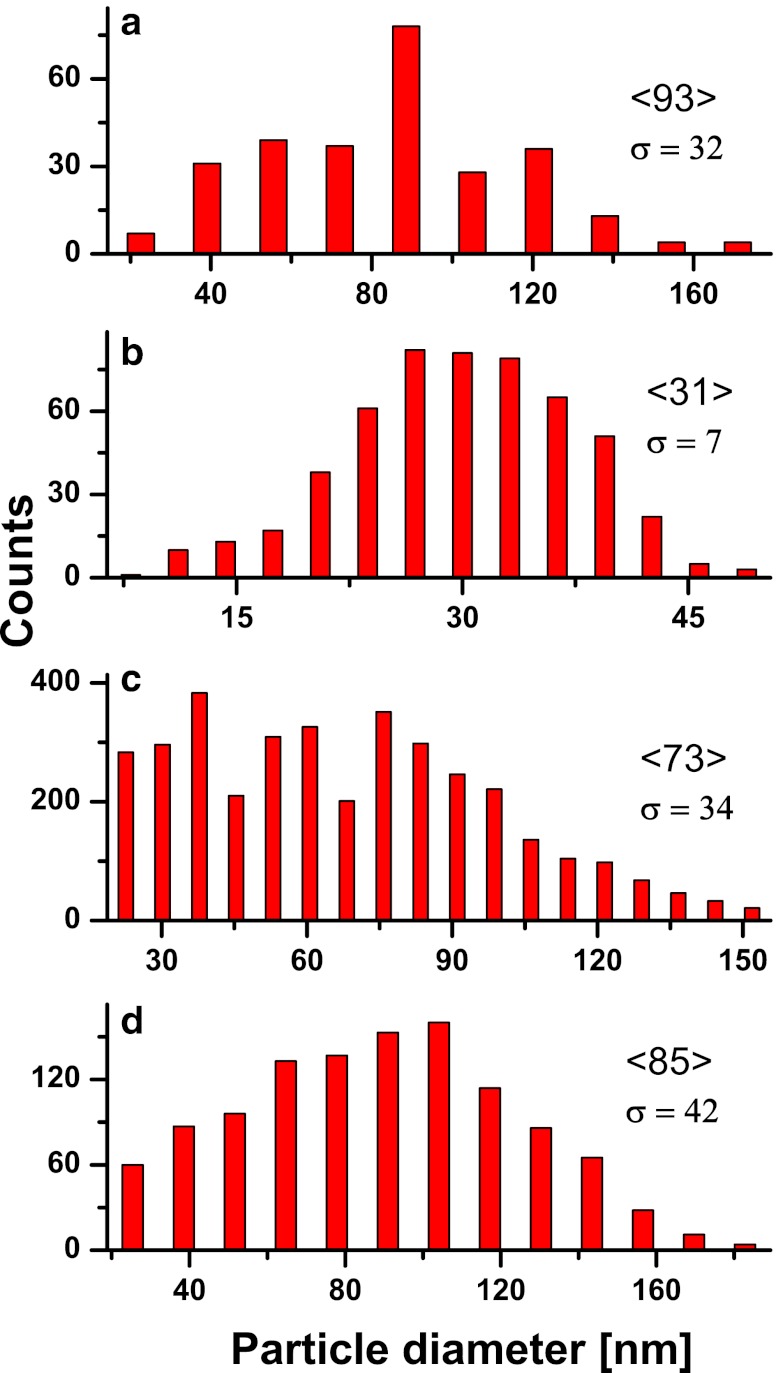
Size distributions of Au nanoparticle structures from Fig. 1; data in diagrams from **a** to **d** with mean values in brackets and standard deviations in percentages correspond to SEM images shown in Fig. 1a–d, respectively

The corresponding insets allow for comparison of their different, nearly homogeneous distributions and also show differences in the inter-particle distances. These three samples of relatively broad size distributions shown in Fig. 2a, c, d, are characterized by the mean size values between ∼70–90 nm. The average inter-particle spacing for large NPs is two to three times the particle size and even larger for smaller ones observed in the images.

The markedly inhomogeneous distribution in Fig. 2a agrees with the sample morphology (Fig. 1a) and results from relatively high laser pulse fluence of 412 mJ/cm^2^ applied to the 10 nm thick film. The particles of larger size appear due to successive annealing pulses and coalescence of the smaller ones which leads to the size redistribution. Mild irradiation conditions and larger pulse numbers lead to normalization and flattening of the size distribution which is accompanied by a decrease of the total particle number (see Fig. 2c, d); and this observation is in agreement with our work reported previously [13, 16]. This result becomes more evident if one takes into account different energy doses applied per unit film volume which are related as 2.06/0.43/0.9/1.5 for nano-arrays in Fig. 1a–d, respectively. For lower values of relative dose the smaller, more uniformly distributed particles are observed (see Fig. 1c); and for a value of 0.43 under certain conditions the closely packed structure shown in Fig. 1b is produced. Consistently, this structure is characterized by uniform particle size distribution (mean value 31 nm) and smallest size deviation of 7 %. In contrary, large irradiation fluencies of the films result obviously in broad size distributions (see Fig. 1a). It is worth noticing that in case of relatively large particles the difference between their size of ∼60–100 nm and the initial film thickness can amount an order of magnitude if only the annealing conditions are properly selected. For samples annealed at fluencies above a certain upper limit (∼0.5 J/cm^2^) decomposition of the thin film into large particulates and irregularly shaped metal islands characterized by sizes of tens of micrometers is observed.

In case of the closely packed structure of smallest size and size distribution (31 nm, 7 %; Figs. 1b and 2b), only a part of the particles reveal circular symmetry while for the rest the moderately irregular or approximately rectangular shapes can be observed. The irregularity of the particle shape is minor, however, similar particles of more elongated shape and also prolate ones characterized by the axial aspect ratio different from unity are known to influence the spectroscopic and optical properties of the structure markedly [17, 18]. Properties of arrays composed of nanorods, and longitudinal nano-assemblies are discussed for plasmonic enhancement due to strong anisotropy of the dielectric field effect which may be induced by linearly polarized light and are of particular importance for efficient light harvesting in the sensing, photocatalysis and photovoltaic applications [9, 19].

**Fig. 3:**
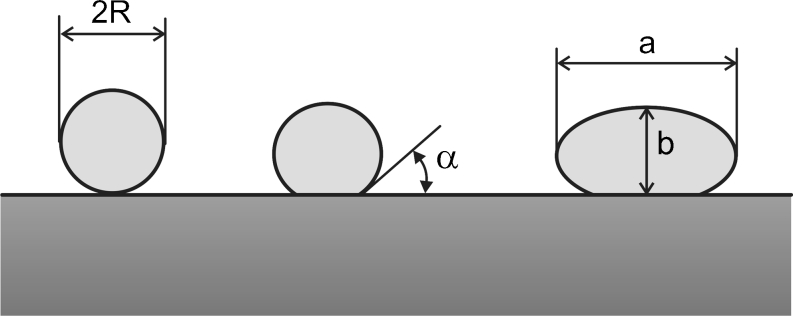
Nanoparticle shapes considered as result of the laser annealing of thin Au films, from left to right: spherical, partially spherical (most realistic case) and spheroidal; the two latter are characterized by the wetting angle α and size aspect ratio a/b

The circular symmetry dominating in the top view of NPs observed in the structures of Fig. 1a–d does not necessarily means that the particle shape effect can be neglected. Recent advances in modeling confirmed by experimental data elucidate the role of shape different from spherical one such as oblate, caps, disks, hemi- and partially spherical, on the peak position and width of the plasmonic resonance [7, 20]. Results of the systematic study of Gupta et al. which analyze the shape concept derived from equilibrium condition of the surface tensions acting on the particle–substrate and particle–ambient interfaces agree with our SEM observation at 45° incidence of laser annealed nano-arrays [7, 14]. It confirms the partial sphere as dominant shape of particles and this is observed independently on the NP size distribution due to laser annealing. This shape corresponds to the case of partial wetting of the substrate by the metal and is characterized by the wetting angle α(α < 180°) which approaches zero with decrease of the metal–substrate interface (weak wetting) and this is schematically shown in Fig. 3. The spherical and spheroidal particles isolated and also incorporated in dielectric medium are subject of modeling based on the Mie theory and calculation of the dipole interaction [7, 20]. However, the recently discussed structures consisting of spheroids (aspect ratio *a*/*b* > 1) does not conform to the aforementioned model assumptions [7]. To our knowledge, the more realistic case of the partial spheroidal shape (see Fig. 3), has not been reported in the literature up to date.

### Absorption Spectra

**Fig. 4:**
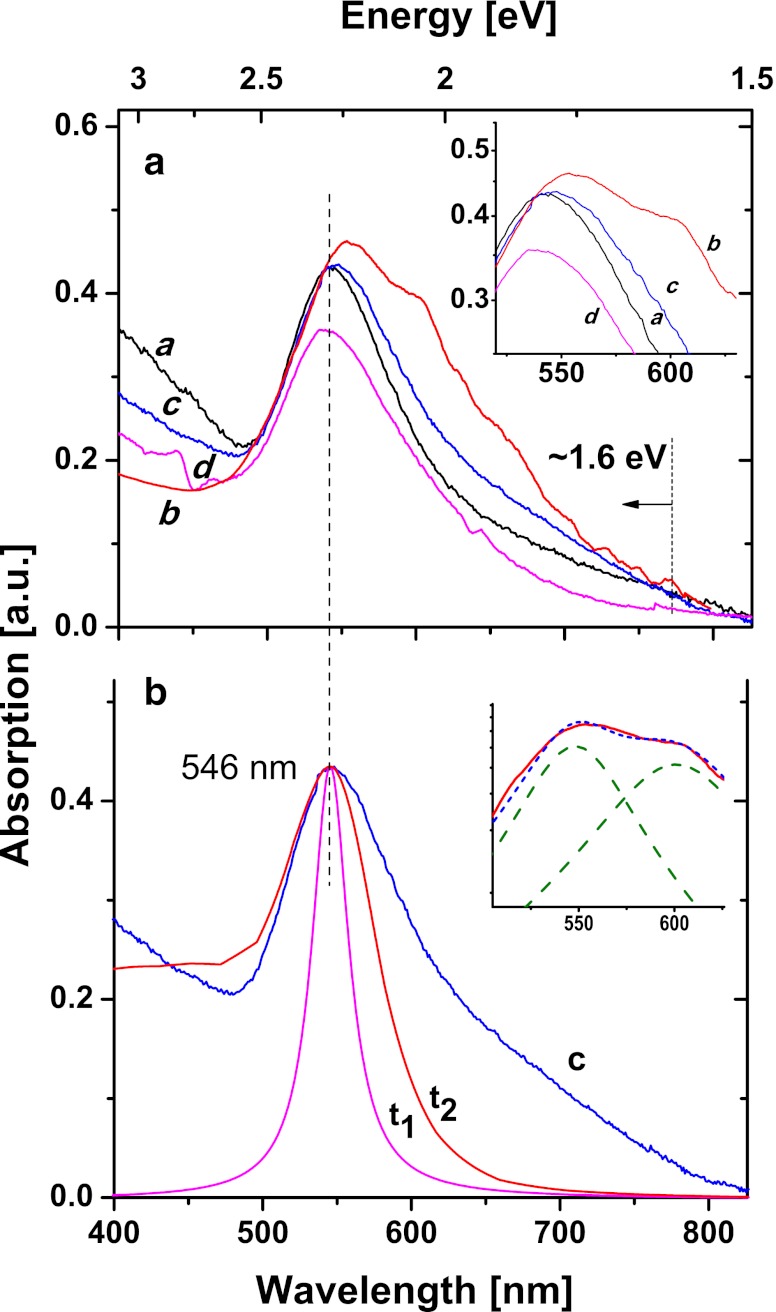
Absorption spectra of the Au nanoparticle arrays on SiO_2_ substrate, profiles are normalized and shifted arbitrarily in height; **a** spectra (*a*, *b*, *c*, *d*) correspond to structures shown in Fig. 1**a**–**d**, respectively; **b** the spectral profile **c** and resonance profiles (*t*_1_) and (*t*_2_) calculated by means of two different methods; *insets* show in **a** details of the resonance maxima, and in **b** components of the double resonance peak of **b**

Measurements of the absorption spectra were performed using the circularly polarized light at normal incidence to the substrate surface. Such selection of the sample excitation conditions has two main advantages. First, it is optimal for structures considered here consisting of NPs which are symmetrical along the irradiation direction. This is justified by a number of theoretical and experimental results [7]. Second, it provides strong resonant response at high signal to noise ratio (S/N) and corresponds to realistic irradiation conditions in a number of applications. Typical absorption spectra of the semi-regular NP structures obtained by UV laser annealing of Au thin films are shown in Fig. 4a.

The experimentally obtained profiles are normalized to equal peak values and then shifted arbitrarily in height relative to each other for clarity. The original, measured peak absorption values amount 12.3 ± 0.4 % for spectral lines (a), (c), (d) and 21.5 % for (b), respectively. These intensities results from the large difference in surface coverage by the AuNPs between structures (a), (c), (d) and the closely packed one (b) (Fig. 1). All structures show absorption growth at excitation above 1.5 eV and its increase at energies of 1.6–1.7 eV corresponds to absorption edge due to onset of the interband transitions (d → p) of Au. The shape and structure of the profile, peak position of the resonant absorption, and structure of its red wing are subject of numerous reports [13, 15]. In short, the nature of the effects lies in the electronic structure of the Au NPs in which the intra- and interband transitions are two main contributions to the observed profile shapes. The first, common to metals occur within the broad conduction band and result in the absorption profile which depends on squared frequency (*ω*) and peaks at *ω*_*p*_ (plasmon resonance frequency). The position of this peak calculated for spherical Au NPs corresponds to excitation around 5 eV which is in-between the known plasma oscillation frequency of Au (8.86 eV) and the experimentally observed values of 2.2–2.4 eV [16]. The second, is specific for Au and is due to the interband transitions between the highest level of the narrow d-band (HOMO) and the conduction band (LUMO) with the first of them at ∼1.6 to 1.7 eV. The mechanism of these d→p transitions substantially determines the strong absorption of the NP structures, however, it is commonly observed for Au surfaces and thin films, too. Moreover, the large polarizability of the Au ionic states leads to a large red shift of the plasmonic resonance. The summary effect of both intra- and interband contributions results in the measured absorption spectra like these in Fig. 4a. From the other hand, the measured resonance position of 2.27 eV (546 nm) compared to the red shifted, calculated one for a single, spherical Au particle is only in part clarified by the particle size, size distribution, packing density in the structure, and by dielectric properties of the surrounding. The literature data on randomly distributed oblate spheroids and also on NPs characterized by a partial spherical shape (α ∼ 25°) indicate that also the particle shape effect should be taken into account here and this conclusion agrees with our SEM observation, too [7]. An extensive review on the geometry factor in tuning of the resonance peak position and profile comprising specific properties of arrays and of individual particles such as: extreme aspect ratio prolate and oblate particles, closely separated particle couples, ultra-thin nanoshells, and also the importance of the concavity factor of individual particle together with the E-field polarization direction is given in [21].

The typical profiles of maxima centered around 2.28 eV (546 nm) represent an experimental evidence of the fact that the intensity of the collective resonance is related to the interband transitions whereas its appearance by the intraband ones. The latter determines also the band position which is, however, strongly modified (red shift) by the polarizability of Au^+^ ions. Because of the observed inter-particle distances, the interactions between NPs are negligible in most cases which results in the same peak positions of spectra (a) (d) and only slightly red-shifted (c) for nano-arrays characterized by NPs of similar shape and size distributions. It agrees with recent results showing that for the semi-regular NP arrays the dipole–dipole interaction vanishes when the ratio of (inter-particle distance/particle radius) > 5. This is different from the case of ordered arrays showing red-shifted resonances scalable with periodicity of the particle’s pattern [22, 23]. For semi-regular structures considered here, the relatively large spectral width (FWHM) of spectra (a, c, d) is characteristic and is also observed in spectrum (b) on Fig. 4a. In this structured absorption profile of a maximum around 2.16 eV (575 nm), two main components centered at 2.06 and 2.25 eV can be separated (see inset in Fig. 4b). Their appearance due to the previously mentioned irregular particle shape observed for this closely packed NP array cannot be excluded. From the other hand, the entire structure in the blue wing of spectrum (b) can in part originate from positive (and destructive) interferences of the strong multipole resonances localized at NP interstices discussed in a number of works [24]. Such resonances appear in the near-field distribution calculated for structure of the measured spectrum (b) and are discussed further in the text.

For quantitative analysis of the experimental data in Fig. 4a, the example of spectrum (c) is briefly discussed below with the size dependent corrections to the Au plasmonic properties taken into account [25]. Implications of the frequency dependent, complex dielectric function of noble metals *ε* ^*^(*ω*) = ε_1_(*ω*) − *iε*_*2*_(*ω*) and the relevant Mie solution of the Maxwell equations yield an expression for the absorption cross-section due to light scattering on small, isolated particles of *R* ≪ λ (*R*, particle radius; *λ* irradiation wavelength) in isotropic media [26]:

1 $$ \alpha \left( \omega \right) = 9{ }{\varepsilon_m}^{{ \frac{3}{2} }}{ }V\frac{\omega }{c}\left( {\frac{{{\varepsilon_2}\left( \omega \right)}}{{{{\left[ {{\varepsilon_1}\left( \omega \right) + 2{\varepsilon_m}} \right]}^2} + {\varepsilon_2}^2\left( \omega \right)}}} \right) $$

where *ω* and *c* are the irradiation frequency and speed of light, *V =* (4*π*/3)*R* ^3^ is the particle volume, *ε*_*m*_, *ε*_*1*_, *ε*_*2*_ are the dielectric constant of the surrounding medium, and the real and imaginary parts of the metal dielectric function, respectively. In the absorption cross-section given by relation (1) the particle dimension *R* appears only as the volume dependent intensity scaling factor. The resonance frequency is given for moderate *ε*_2_ values by the condition *ε*_1_(*ω*) *= −* 2*ε*_*m*_. The need for introduction of the size and quantum size dependence into (1) follows from comparison of the FWHM values of spectrum (c) typical for the considered, rarely packed NP structures with the calculated ones of profiles (*t*_1_) and (*t*_2_) in Fig. 4b. These are calculated for *R* = 36.5 nm using relation (1) for (*t*_1_) and the MiePlot numerical package for (*t*_2_) and are shown normalized to (c).

In frames of the Drude model, the FWHM value is a measure of damping *Γ* of the free-electron oscillations in the isolated Lorentz spherical cavity, and the dielectric function can be written in the form:

2 $$ \varepsilon \left( \omega \right) = 1 - \frac{{{{\omega }_{p}}^{2}}}{{\omega (\omega + i\Gamma )}} $$

where ${\omega_p} = n{e^2}/{m_e}$ is the plasma oscillation frequency of electrons, with the free electron density *n*, the electron charge *e* and effective mass *m*_*e*_. The maximal cross-section follows from (1) at resonance, i.e., for ${\omega_0} = {\omega_p}{\left( {1 + 2{\varepsilon_m}} \right)^{{ - 1/2}}}$ and corresponds to conditions of pure radiative damping of the free electron oscillations. The size-dependent corrections proposed in the literature and of practical meaning base on the *Γ*(*R*) dependence:

3 $$ \Gamma (R) = {{v}_{F}}\left( {\frac{1}{{{{l}_{0}}}} + \frac{A}{R}} \right) $$

in which *v*_*F*_ is the Fermi velocity (1.4 × 10^6^ m/s for Au); *l*_0_, the electron mean free path; and *A*, the proportionality constant, dependent on scattering processes taken into account. For *A* values in the range of 0.1−2 have been theoretical justified and allow for reasonable fitting of the experimental half width data in case of a small, single particle [25, 27]. In relation (3) only the second term $A{v_F}/R$ is size dependent and represents the scattering rate on the particle surface. It sets also the physical limit of *l*_0_ due to NP dimension. The exemplary calculation of (*t*_1_) with *l*_0_ = 20 nm and frequency dependent components *ε*_1_, *ε*_2_ for Au results in a correct peak position for *ε*_*m*_ = 3.65. The value of *l*_0_ taken from measurements of Theye [28] is smaller than the mean free path of 40 nm deduced from electrical measurements in bulk gold and this accounts for additional electron scattering on the particle interface [29]. In case of (*t*_2_) with the software-predefined complex value of *ε* ^*^ the profile shows peak at 546 nm for slightly elevated *ε*_*m*_ = 1.9. In both cases, the FWHM values account only in part for the measured line broadening (see Fig. 4b). It follows from a series of fitting trials that coincidence of the calculated FWHM with experimental one can be obtained for much smaller *R* values, however, it requires larger *ε*_*m*_ (and *l*_0_) in order to obtain the correct peak position. This indicates on the need of completion of the existing models with additional size correction which take into account specific properties of the semi-regular structures of size-distributed NPs of partial spherical shape on substrates. Note that in this discussion, the substrate influence and interband transitions are not taken into account.

The damping processes described by relation (3) result in shortening of the coherent lifetime of the plasmon oscillation, resonance band broadening, and limiting of the enhancement of the local field. The origin and amount of the plasmon damping depend strongly on properties and quality of the NP structures, and the relevant data can be extracted from spectral profiles of the resonant absorption characterized by the dephasing time ${{T}_{2}} = 2\hbar /\Gamma$. The size-dependent dephasing time is given by the expression:

4 $$ \frac{1}{{{T_2}}} = \frac{1}{{{T_r}}} + A\frac{{{v_F}}}{R} $$

where the bulk-specific, purely ratiative relaxation time *T*_*r*_ reported up to date for Au is equal to 18 fs and can be used as reference value for estimates of the quantum efficiency of the resonant scattering [17]. The related *l*_0_ value of 12.5 nm agrees with data obtained by Theye [28]. For gold NPs, the decrease of *T*_2_ with increasing particle diameter and strong dependence on the particle shape is reported [17, 30]. The dephasing time values calculated from experimental spectra in Fig. 4 obtained for similar, rarely packed structures (a), (c), and (d) are equal to 2.8 ± 0.2 and 1.83 fs for the closely packed one (b). These data correspond to particle size larger than 100 nm which is in disagreement with the average dimensions estimated from histograms. Also, the values of 10 and 5.2 fs calculated from profiles (*t*_1_) and (*t*_2_) show that some damping components characteristic for the semi-regular structures are missing in the modeling and an additional size dependent correction is required in the second term of relation (4). The primary reason of and contribution to the difference between modeling and experiment can be ascribed to the specific of the nano-array production by pulsed laser irradiation of thin Au films. The resulting size- and inter-particle distance distributions and also the particle aspect ratio and shape are represented in the model by one variable which depends on the average particle size. In this way, the constructive effect of the crystalline phase content (crystalline Au reveals weaker resonance damping than amorphous one) and also the dependence of the *ε*_1_(*ω*) on the inter-particle distance (the particles behave less metallic with increasing separation) are not taken into account [31]. Moreover, the constructive and destructive components of the collective resonant response of the structure require further study of the responsible mechanisms in order to be included in the modeling. Nevertheless, our experimental data are consistent with these reported recently of the spectrally broad resonances of similar lifetimes observed for the random, vapor-deposited Au NP structures and also for annealed thin films characterized by particle size distributions [7, 20].

### Near-Field Intensity Distribution

**Fig. 5:**
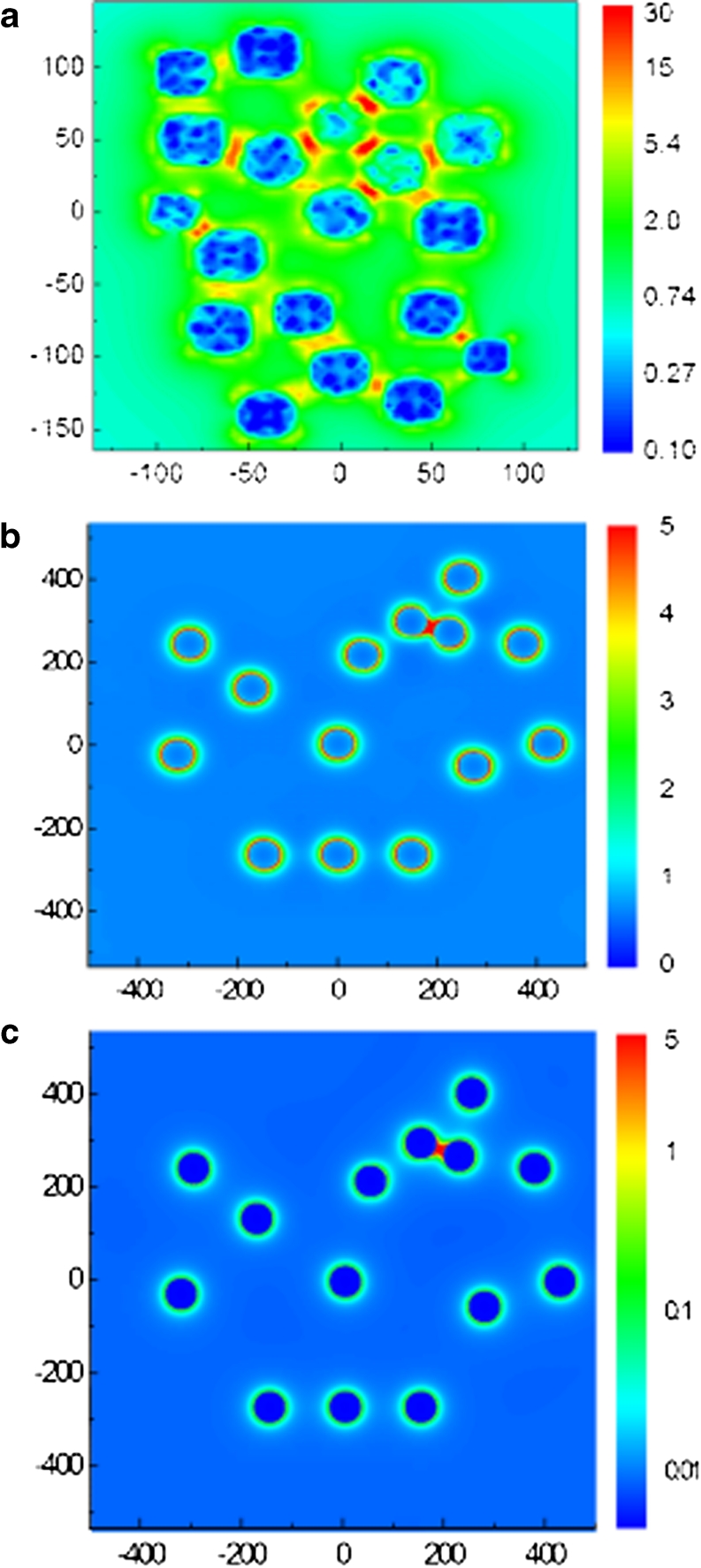
Near-field intensity distributions in the vicinity of Au nano-arrays on SiO_2_, calculated for structure geometry adopted from SEM image in Fig. 1b, for assumed particle size 31 nm (**a**) and from Fig. 1c for particle size 73 nm (**b** and **c**); intensities (logarithmic scale) are related to constant irradiation field of 1 V/cm^2^ at wavelength 785 nm in **a**, **c**, and at 514 nm in **b**; array dimensions are given in nanometers

The field enhancement distributions are obtained from the finite difference time domain (FDTD) numerical calculation performed for the semi-regular structures. The geometries are taken from SEM images of the experimentally obtained nano-arrays. The areas simulated by the numerical model are marked by squares in Fig. 1b and c. These structure fragments are used to simulate a system of spherical Au NPs on the glass substrate in which the coordinates (*x*, *y*) of the 19 and 14 particles of diameters of 40 and 73 nm are reproduced from Fig. 1b and c, respectively. The particle dimensions are averages assumed from the corresponding size distributions in Fig. 2. The frequency dependent dielectric function of the Au NPs is taken from [14]. The calculated intensity distributions of the near field│*E* ^2^│in a plane distanced by the particle radius *R* and parallel to the substrate surface are shown in Fig. 5. The enhancement is related to the squared input value of *E* = 1 V/m of the incident, plane light source illuminating orthogonally the surface at wavelengths of 785 or 514 nm which are typical for Raman spectrometers. For the closely packed structure (Fig. 1b), the higher light enhancement is calculated in case of excitation at 785 nm than at 514 nm (Fig. 5a) [14]. It results from the red shift of the resonant absorption of the array structure compared to the case of a single particle of the same average diameter for which the dipolar resonance peak is reported at approximately 550 nm [11]. The intensity maxima (“hot spots”) observed in the distribution areas of strongest enhancement is located between the most closely separated nanoparticles. Numerous intensity peaks form a partially symmetrical pattern due to semi-regularity of the closely packed NP structure. The spatial intensity distribution determines the multipolar character of the plasmonic enhancement and can lead to differences in the resonant response at different excitation energies. The particle shape, size, and size distribution, and also inter-particle distances are main contributions to the observed effect. This conclusion is in agreement with recent literature data on disordered plasmonic structures [7, 20]. It is also in accordance with the structured, broad resonance profile of the experimental absorption spectrum (b) shown in Fig. 4.

The near-field distributions in Fig. 5b, c calculated for largely separated particles of average diameter of 73 nm show maximal field intensities smaller by an order of magnitude than in case of the closely packed structure. It is mainly the consequence of the structure geometry and also relatively low scattering efficiency on particles of that size. The field distribution is dominated by the dipolar effect localized on individual particles. Because of large inter-particle distances the coupling between the local dipole fields cannot build up and the enhancement due to field in the inter-particle areas is practically negligible. The field distribution provides a direct indication that in case of this structure the resonant response due to light scattering, i.e., properties of the absorption band are similar to that of a single particle. Indeed, comparison of the field distributions (b) and (c) in Fig. 5 shows that excitation at 514 nm is more efficient than at 785 nm. This, together with the position and width of the absorption band in Fig. 4 clarifies the preference of the excitation at 514 nm and is in accordance with data reported for the resonant response of periodic and randomly distributed NP arrays produced by thin film annealing, ion beam lithography, and colloidal ones [19, 20].

### Far-Field Enhancement

**Fig. 6:**
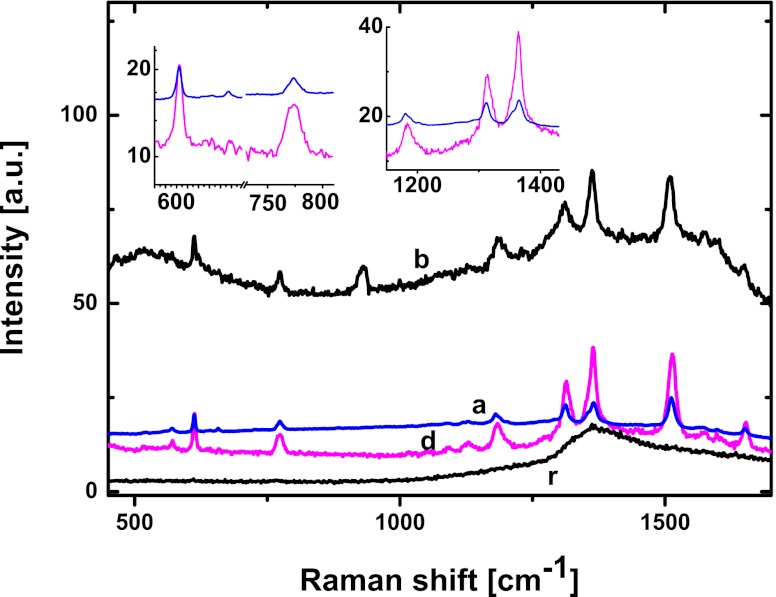
Raman spectra (*a*, *b*, *d*) of R6G deposited on semi-regular arrays of Au NPs shown in Fig. 1a, b and d, respectively, and on glass (r); excitation at 785 nm; *insets* show spectral regions of surface enhanced signal

Measurements of the μ-Raman spectra were performed to analyze the relation between the morphology of the semi-regular nano-arrays, the near-field distribution of the light intensity induced by the plasmonic resonance and the SERS observed in the far field. The spectra (a, b, d) obtained for nano-arrays and also reference spectrum (r) of the SiO_2_ substrate all covered with dried solution of 0.09 M R6G are shown in Fig. 6.

The direct comparison of spectra: (r) of the glass substrate, and (b) of closely packed NP structure, shows essential intensity difference and the Raman peaks in (b) at 610^1^, 773, 1,180, and 1,363 cm^−1^ due to strong, surface enhanced signal. It is obvious that the intensity peaks in spectra (a, d) recorded for rarely packed structures of similar geometry are typically of lower intensities.

It should be mentioned, that spectra of the same samples (a, d) recorded for excitation at 514 nm reveal markedly lower Raman signal. Such observation seems surprising in view of the stronger field enhancement obtained from the FDTD modeling at this wavelength compared to 785 nm. The most probable explanation can be the following. First, the surface coverage by AuNPs is relatively low and lies between 12 and 28 % while the R6G covers the entire surface of the nano-array. As a consequence, most of the incident radiation does not contribute to scattering on Au particles and is absorbed by the organic molecules. Moreover, this absorption is rather efficient under excitation at 514 nm partially overlapping with the R6G absorption band centered at 530 nm. It competes with the broad absorption (peak position, 546 nm; FWHM, ∼110 nm) of Au NP arrays investigated here. In result, it decreases the irradiation intensity of the Au particles and the contribution of the resonant scattering decreases accordingly, which lead to lower values of the near-field enhancement. This can clarify why for surface covered with R6G the smaller Raman signal is measured for excitation at 514 nm compared to 785 nm. In the case of 785 nm, the plasmonic absorption intensity dominates over the very weak R6G absorption at this wavelength and this lead to higher enhancement of the Raman signal. Conversely, in the absence of R6G coverage on the nano-array surface the excitation at 514 nm, i.e., in the blue wing of the plasmonic band is more efficient than in the red one (785 nm) thus showing higher enhancement.

Comparison of the calculated intensity distributions of Fig. 5 and the Raman data in Fig. 6 confirm that under given excitation the coincidence of the near-field resonant enhancement with the surface enhancement observed in the far field via Raman spectra is sensitive to the relative surface coverage by the Au NPs. This effect can be tuned by the nano-array properties and also a threshold value can be engineered for different excitations. The resulting switching concept requires, however, further investigation.

## Conclusion

Optical properties of the rare- and close-packed gold nano-arrays produced by the UV laser annealing of thin films were investigated by means of the SEM microscopic observation, measurements of the absorption and Raman spectra, and by numerical modeling of the near-field intensity distributions. It has been shown that nearly homogeneous particle distributions can be prepared by careful selection of the initial film thickness and annealing conditions. The partially spherical shape of the particles contributed to broadening of the absorption band, became irregular for densely packed arrays and provided marked difference in the optical response between the rare- and close-packed structures. The absorption spectra revealed broad, structured resonances of intensities strongly dependent on the NP packing density and distribution. It was the result of the inevitable randomness in the structures in which all particles differ slightly in the resonance energies due to different interaction with the neighboring ones. This was also confirmed by the calculated field distributions which revealed “hot spots” for dense structures while individual dipolar resonances dominated in arrays with large inter-particle distances. Comparison of experiment with data calculated from two different models confirmed that description of optical properties of semi-regular arrays of partially spherical NPs can be provided by modification to the known, size-dependent correction of the dielectric function. This was found possible by simplified approach using relatively large values of the refractive index (*n* > 3.5) and mean free path limits and indicated on the need of further systematic study. The broadband resonances of lifetime reduced to ∼3 fs and position selected by the array preparation indicated on capacity in nonlinear applications such as spectral tuning of light harvesting devices [32], ultra-sensitive detection (SERS), and all optical switching. Finally, it was shown that semi-regular Au NP arrays produced by laser annealing of thin films are advisable because of controllable and cost-effective preparation and also structural and chemical stability which is of crucial importance for the industrial scale plasmonic technologies.
